# Covariates of corticotropin-releasing hormone (CRH) concentrations in cerebrospinal fluid (CSF) from healthy humans

**DOI:** 10.1186/1471-2202-5-58

**Published:** 2004-12-17

**Authors:** James N Baraniuk, Hilda Maibach, Gail Whalen, Daniel J Clauw

**Affiliations:** 1 Division of Rheumatology, Immunology and Allergy, Room GL-002, Lower Level Gorman Building, Georgetown University, 3800 Reservoir Road, NW, Washington, DC 20007-2197; 2 Center for the Advancement of Clinical Research, The University of Michigan, Ann Arbor, MI

## Abstract

**Background:**

Define covariates of cerebrospinal corticotropin-releasing hormone (CRH) levels in normal humans. CRH_CSF _was measured in 9 normal subjects as part of an intensive study of physiological responses stressors in chronic pain and fatigue states. CRH_CSF _was first correlated with demographic, vital sign, HPA axis, validated questionnaire domains, baseline and maximal responses to pain, exercise and other stressors. Significant factors were used for linear regression modeling.

**Results:**

Highly significant correlations were found despite the small number of subjects. Three models were defined: (a) CRH_CSF _with blood glucose and sodium (explained variance = 0.979, adjusted R^2 ^= 0.958, p = 0.02 by 2-tailed testing); (b) CRH_CSF _with resting respiratory and heart rates (R^2 ^= 0.963, adjusted R^2 ^= 0.939, p = 0.007); and (c) CRH_CSF _with SF-36 Vitality and Multidimensional Fatigue Inventory Physical Fatigue domains (R^2 ^= 0.859, adjusted R^2 ^= 0.789, p = 0.02).

**Conclusions:**

Low CRH_CSF _was predicted by lower glucose, respiratory and heart rates, and higher sodium and psychometric constructs of well being. Responses at peak exercise and to other acute stressors were not correlated. CRH_CSF _may have reflected an overall, or chronic, set-point for physiological responses, but did not predict the reserves available to respond to immediate stressors.

## Background

Corticotropin – releasing hormone (CRH) plays a major role in regulating the hypothalamic – pituitary – adrenal (HPA) axis, acute responses to stressors, and other neurological functions [1]. The cerebrospinal concentrations of CRH (CRH_CSF_) and neuropeptide Y (NPY), another important neuropeptide involved in pain, autonomic and stress responses [2,3], were measured in 9 normal humans to better understand these functions. Subjects were studied as part of a large scale investigation of subjective and objective (functional magnetic resonance imaging) responses to pain testing, exercise, and other stressors [4]. Subjects completed questionnaires that assessed pain, anxiety, depression, coping skills, and other psychometric variables. Normal control, fibromyalgia, [5], chronic fatigue syndrome [6], and veterans of the first Persian Gulf War [7] were included. This pilot investigation focuses on the statistical strategy used to analyze the control population (n = 9), and to develop methods for effective evaluation of patient populations. Three multiple linear regression models [8] were defined that predicted CRH_CSF _based on (a) metabolic, (b) autonomic, and (c) psychometric measures.

## Results

### Demographics

The mean age for the group was 35.2 yr (95% C.I.: 30.5 to 39.9) years. There were 2 females, 5 African-Americans, 3 Caucasians, and 1 Caucasian Hispanic (TABLE 1). These subjects were a very normal and healthy group based upon their histories and the strict exclusion criteria.

### NPY_CSF_

Mean NPY_CSF _was 121.9 (87.8 to 156.0) pg/ml. NPY did not correlate with any variable.

### Variables correlated with CRH_CSF_

**Figure 1:**
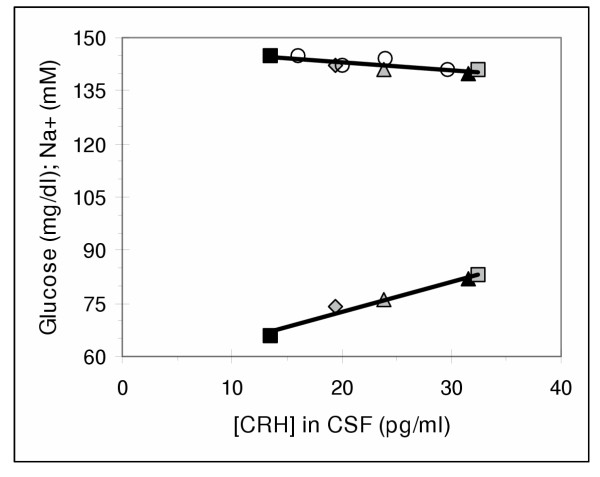
Relationships between CRHCSF, plasma glucose, and serum sodium. Data from single individuals are shown with identical symbols (e.g. solid black squares on left). Open circles indicate sodium without corresponding glucose values. The linear regression lines for CRHCSF with sodium (top line) and glucose (bottom line) gave explained variances (r2) of 0.66 and 0.97, respectively.

Significant covariates of CRH_CSF _could be grouped into: metabolic, autonomic function, and perceptional and cognitive functions (TABLE 3). Serum glucose was positively, and sodium negatively, correlated with CRH_CSF _(FIGURE 1). Explained variances (R^2^) were 0.97 and 0.66, respectively. Glucose and sodium were also negatively correlated (R^2 ^= 0.85). Resting norepinephrine levels at 2 time points and heart rate at 4 time points were colinear and positively correlated to CRH_CSF_.

CRH_CSF _was negatively correlated with the Holter monitor-derived measure of log heart rate summed for the daytime, and the threshold temperature causing an initial, mild sensation of burning pain (Stressor I). The latter indicated that subjects with higher CRH_CSF _perceived the burning pain of the cutaneous forearm hyperthermic stimulation at a lower threshold temperature than their peers. This may indicate an increased sensitivity to nociceptive stimuli. However, there was no correlation with deep pressure – induced pain. Negative correlations were also found with the SF-36 Vitality and SES Manage Symptoms domains. High scores were normal for these questionnaires, with lower scores indicating dysfunction.

### Effects in males

All parts of the study were completed by at least 5 males. Analysis of the male subgroup gave some information about the role of gender. The pattern of significant covariables was different from the total group (TABLE 4). Respiratory rate was the only vital or physiological sign related to CRH_CSF_. Perceptions of vulnerability, physical functioning and self-efficacy were more highly related.

### Statistical models

Sets of the significant metabolic, autonomic, and perceptual variables were grouped and analyzed by 3 linear regression models in order to detect significant relationships between independent variables and CRH_CSF_. The 3 models had high significance levels (TABLE 5). A metabolic model related CRH_CSF _to glucose and sodium. An autonomic model linked CRH_CSF _to resting respiratory and heart rates. The optimum perceptual model predicted CRH_CSF _based on SF-36 Vitality and MFI Physical Fatigue. The latter 2 domains were not the same as those in TABLES 3 and 4 because many of these variables were co-linear or surrogates of one another. This was reinforced by the similarity of R^2 ^and p values where these domains were substituted for Vitality and Physical Fatigue. These results were remarkable because it is very unusual for models with such small numbers of observations (n ≤ 9) to be so significant (p < 0.02). The explained variances were very high (R^2 ^> 0.85) suggesting that the factors may be causally connected.

CRH_CSF _did not correlate with variables associated with maximum exercise, heat – and pressure – (dolorimetry) induced pain, or other rapid onset stressors (TABLE 1).

## Discussion

Despite the small numbers of normal subjects in this pilot investigation, the data, the explained variances for relationships, and final 3 models predictive of CRH_CSF _were highly significant. The models were of importance, since they reflected the specific neurological functions of this neurohormone. CRH and NPY were co-expressed in the hypothalamus [2], but NPY_CSF _did not correlate with CRH_CSF _or any other variable.

CRH and the hypothalamic-pituitary-adrenal axis maintain numerous systemic functions. Our metabolic model showed a tightly correlated relationship between 2 pm serum glucose, sodium and CRH_CSF_. Relatively higher CRH_CSF _levels were associated with elevated serum glucose levels. When glucose is elevated, it is pumped into cells along with sodium ions [9]. In our model, this may have been reflected by the reduced serum sodium concentrations (FIGURE 1). These measurements were taken at different times, suggesting that the CRH_CSF _set a long-term operating range for this system. The variables were interrelated. CRH_CSF _in the low normal range was inferred from a low glucose and relatively high sodium. Other reports also suggest a role of CRH and energy balance [10].

Neuroendocrine responses such as these rely solely on CRH type 1 (CRH1) receptors and the HPA axis [11]. The other CRH receptor gene, CRH2, has 3 splice variants (α, β and γ) but only CRH2α is expressed in the brain. CRH1 and CRH2α receptors have nonoverlapping distributions, but mediate many similar defensive behaviors suggesting that they act in parallel neural circuits. Different stressors may act by separate circuits and have distinct feedback and control systems [11-13]. CRH1 receptors in the central nucleus of the amygdala may participate in conditioned fear responses [12]. These CRH neurons may project to the hippocampus and CRH1 receptors in the locus ceruleus to induce defensive behaviors and autonomic reflexes [13,14]. Dorsal raphe nucleus neurons may release CRH that acts on inhibitory CRH2α receptors in the lateral septum [15]. Neurons from the lateral septum tonically inhibit periaqueductal grey regions that induce similar defensive behaviors. These nuclei are probably additional sources of CRH in the CSF.

Resting respiratory and pre-exercise heart rates and CRH_CSF _were positively correlated. The statistical model indicated that a low CRH_CSF _was predicted by low respiratory and heart rates. Respiratory and cardiac functions are rigorously controlled by brainstem and other nuclei that integrate incoming signals of plasma O_2_, CO_2 _and H^+ ^concentrations, activity needs, anxiety and other stressors. Efferent cardiovascular and other autonomic reflexes are modulated by CRH in man [16]. These central nervous system effects may be due to, or highly correlated with, CRH_CSF_. This is supported by studies in mice that genetically overexpress CRH. They develop chronic stress – like autonomic and physiological alterations [17]. Stressors acting via conditioned fear responses may involve CRH1 receptors in the central nucleus of the amygdala. Some of these CRH neurons project to locus ceruleus neurons [17] that activate autonomic reflexes and defensive behaviors such as "freezing" (immobility) in rodents [14]. An example of this valuable defense would be the freezing of prey in the presence of a predator. Immobility would allow the prey's camouflage to blend into the surroundings without generating motion – induced visual cues for the predator. In humans, excessive, aberrant or dysregulated manifestions of defense behaviors such as freezing may contribute to the immobility, inertia, or even catatonia that contribute to the clinical picture of depression [1].

Extrapolation of these concepts suggests that elevated CRH may be related to anxiety, depression, or other disorders associated with chronic stress responses. If so, then lower, but normal, CRH_CSF _should be present in persons lacking these stressor states. This was supported by the negative correlations of CRH_CSF _with scores for the SF-36 Vitality and Change in Health, Self Efficacy Scale Manage Symptoms, MIQ Vulnerability, and MFI Physical Functioning domains. Each scale has an idealized "normal" end of the range of scores. Deviation towards either higher (MFI) or lower (SF-36) ends of the scales provides an estimate of dysfunction. For each of these domains, the lower CRH_CSF _were associated with more normal scores, while higher CRH_CSF _was associated with scores that were beginning to shift away from the normal pole of each scale. These trends were further supported by the statistical model where the optimum covariates of CRH_CSF _were SF-36 Vitality and MFI Physical Fatigue domains. The model predicted that CRH_CSF _would be in the low normal range when Vitality and Physical Fatigue domain scores were high (normal). Taken together, these results confirm the consistent physical status, mental coping skills, and general health of these subjects.

Studies of intraventricular CRH injection in primates support our findings [33]. The CRH diffused to brain regions that led to 3 types of behavioral changes. Externally oriented behaviors such as locomotion and environmental exploration were significantly decreased. Anxiety – related self – clasping was increased. Depression – like behaviors of avoidance of social contact, huddling, slouching, and wall facing were seen only in social settings. There were high interindividual differences in responses, but a key element was the social context in this study. This social context is lacking in rodent studies where animals are typically studied in isolation.

## Conclusion

This small but intensively studied group of normal humans demonstrated surprisingly robust relationships between CRH_CSF _and metabolic, autonomic, and psychometric measures. These are novel findings in humans, but are consistent with data on CRH in acute and chronic stress models, and proposed CRH neural circuits. It will now be of great interest to contrast these statistical models identified for normal subjects with the other chronic pain and fatigue patient subsets to determine if CRH_CSF _correlates with different sets of variables.

## Methods

### Subjects

Nine normal, healthy subjects (2 females) gave informed consent for this paid, Institutional Review Board – approved protocol. They had a comprehensive screening evaluation to exclude: severe physical impairment, morbid obesity, autoimmune/inflammatory diseases, cardiopulmonary disorders, uncontrolled endocrine or allergic disorders, malignancy, severe psychiatric illnesses (e.g., schizophrenia, substance abuse), factors known to affect the HPA axis or autonomic function (cigarette smoking, daily intake of caffeine exceeding the equivalent of 2 cups of coffee), or medication use. Subjects had a history and physical examination, were administered the Structured Clinical Interview for the Diagnostic and Statistical Manual of Mental Disorders, fourth edition (DSM-IV) (SCID II) [19,20], Composite International Diagnostic Interview (CIDI) [21,22] and Center for Epidemiologic Studies Depression scale (CESD) [23] to detect psychiatric co-morbidities, and completed the self – report Short Form 36 (SF-36) [24,25], Self Efficacy Scale (SES) [26], Meaning of Illness Questionnaire (MIQ) [27], and Multidimensional Fatigue Inventory (MFI) [28].

### Study protocol

Subjects were admitted to the G-CRC on the evening of Day 1. An 18-gauge catheter was inserted antecubitally and infused with normal saline at 50 ml/hr. A Holter monitor was attached to monitor autonomic regulation of cardiac rhythm [29-31]. Upon awakening, or at 6:30 am, subjects stayed in bed until they had "pre-awakening" blood tests drawn. Breakfast was served between 7:00 and 8:00 a.m. and baseline Day 2 blood samples drawn at 8:30 a.m. Vital signs, questionnaire responses, and other blood samples were drawn from the catheter to assess HPA axis and stress system responses before, during and after Day 2 stressors. Pain responses were tested by pressure applied to each subject's thumbnail and heat to the forearm applied in random staircase testing paradigms (Stressor I) [32,33]. Stressor II was a series of cognitive challenges including the Benton Visual Retention Test of visual-spatial memory; Digit Span recitation; and Pig Latin, a test of verbal working memory [34]. Lunch was served, followed by a 2 to 2.5 hours rest break to allow post-prandial catecholamine levels to reequilibrate. At 2 pm, serum glucose, electrolytes, plasma catecholamines and other analytes were measured. Subjects then squeezed an isometric hand grip dynamometer to test autonomic function and muscular fatigue [35] (Jamar, Sammons Preston, Bollingbrook, IL) (Stressor III). After 30 min of rest, they had a sub-maximal exercise test on an electronically-braked cycle ergometer (Sensormedics, Yorba Linda, CA). The test was graded in 3 min stages and ended when the subjects' heart rates reached 85% of their age-predicted maximum (Stressor IV). Lumbar punctures were performed 30 min later (approximately 4 pm) (Stressor V). Subjects sat while sterile technique was used to prepare the skin over the L4–L5 lumbar region, infiltrate the subcutaneous and deep tissues with 2% lidocaine, and insert a 22G spinal catheter. CSF was collected as 3 to 4 aliquots of about 2 ml each. Catheters were withdrawn and subjects allowed to rest in their preferred position for 30 min.

### Lumbar punctures and CRH and NPY radioimmunoassays (RIA)

Tubes of CSF were immediately placed on ice and then centrifuged at 4°C. The supernatants were rapidly frozen at -80°C. Tube 2 or 3 was removed from the freezer and thawed at 4°C. Peptides were extracted by precipitating high molecular weight proteins by adding an equal volume of 100% ethanol, 0.1 M acetic acid, 0.2% sodium bisulfite [36,37]. The supernatant was dried (SpeedVac, Thermo Savant, Holbrook, NY), resuspended in phosphate buffered saline with 1% bovine serum albumin (RIA buffer; Peninsula Laboratories, Inc., San Carlos, CA.). Samples and standard amounts of each peptide were aliquoted and peptide specific rabbit antibodies added. After overnight incubation at 4°C, I^125^-CRH or -NPY was added, tubes gently vortexed, and again incubated overnight. Goat anti-rabbit antibodies were added, tubes incubated, and immune complexes precipitated by centrifugation. Radioactivity was counted for the standards, and the concentrations for each sample interpolated from the standard curves. Spiking CSF with fixed amounts of I^125^-peptides and performing the RIA shifted the curves to the left by the anticipated concentrations. Standard curves were reproducible to within 10%.

Plasma catecholamine levels were measured by HPLC (Mayo Clinic Laboratories, Rochester, MN).

### Statistical methods

All the data from the provocation studies, questionnaire domains, blood work, and neuropeptide analysis were entered into a SAS spreadsheet (SAS, Carey, NC) using sequential hand or scanner entry followed by data checking routines.

Means with 95% confidence intervals were reported.

Simple correlations between our outcome measure (CRH_CSF_) and other objective and subjective patient variables were conducted as an exploratory tool. Given the small sample size (n = 9), both parametric and non-parametric correlations were used to evaluate the robustness of the correlation coefficients. Partial and intra-class correlations examined the structure of the relationships between CRH_CSF _and the independent variables. Original data were always checked to make sure that correlations were not spurious, due to outliers, colinearity, or sets of virtually identical scores.

Our data included a very wide range of covariates which spanned the physical and psychological attributes of the patients. Scatter plots were used to determine the response slopes with CRH_CSF_. Independent variables with flat slopes or high scatter that did not generate correlations with CRH_CSF_, that were highly correlated with each other (collinear), or that had < 5 recorded values were excluded from further analysis.

The remaining suitable independent variables fell into 3 categories: metabolic, autonomic and psychometric function. Three 3 separate linear regression models [8] were constructed to predict CRH_CSF_, the dependent variable. Separate models were required because of the low degrees of freedom available for the analyses, and to ensure that the explained variance (R^2^) was not inflated due to overloading the model with potentially collinear variables. Variance inflation factors and tolerance values were incorporated to optimize the selection of the most independent combinations of variables that also maximized the R^2 ^and p values. The linear regression procedure took into account the multiple comparisons for each model.

## Abbreviations

CESD, Center for Epidemiologic Studies Depression scale; CFS, Chronic Fatigue Syndrome; CIDI, Composite International Diagnostic Interview; CRH, corticotropin-releasing hormone; CSF, cerebrospinal fluid; MFI, Multidimensional Fatigue Inventory; MIQ, Meaning of Illness Questionnaire; NPY, neuropeptide Y; R^2^, explained variance; SCID II, Structured Clinical Interview for the Diagnostic and Statistical Manual of Mental Disorders, fourth edition (DSM-IV); SF-36, Short Form 36; SES, Self Efficacy Scale

## Authors' contributions

JNB directed the RIA studies, correlation of clinical variables, and wrote the manuscript. HM confirmed the accuracy of the study results by double data entry methods, vetted the SAS database, performed the statistical analysis, and co-wrote the manuscript. GW performed the CRH and NPY assays and maintained the repository of these samples. DJC was the Principal Investigator for the overall study and helped review the draft and final manuscript.

## Figures and Tables

**Table 1:** Physiological data for each individual stratified by CRH_CSF_

Age (yr)	CRH_CSF _(pg/ml)	Sex	HR^a^	HRV log p (day)^b^	Heat Score^c^	Tender points (#)	Pressure threshold (kg/cm^2^)	Epi^d^	Norepi^e^
48	32.4	M	76	7.0039	37.7	0	8.32	0	330
31	31.5	F	76	7.128	37.8	0	2.90	0	
29	29.7	M	73	7.2561		0		13	
35	24	M	68	7.3232	40.5	1	10.11	14	430
31	23.8	M	89	7.2077		0	9.78	20	202
43	19.8	M	48	7.6277	43.9	1	8.67	16	223
25	19.4	M	77		41.4	0	4.76	10	100
36	16	F	76	7.5555	48.8	0	11.71	0	199
39	13.5	M	59	7.3973	43.6	4	7.48	13	182

HR^a^, heart rate; HRV log p(day)^b^, log10 of summed daytime heart rate intervals by Holter monitor; Heat Score^c^, unpleasantness rating given for the first sensation of heat; Epi^d ^and Norepi^e^, plasma concentrations of epinephrine and norepinephrine immediately prior to bicycle exercise

**Table 2:** Psychometric data for each individual stratified by CRH_CSF_

Age (yr)	CRH_CSF _(pg/ml)	Sex	SF36 CH^a^	SF36 Vitality	CESD	SES manage symptoms
48	32.4	M	25	35	6	5.2
31	31.5	F	50	45	10	7.5
29	29.7	M				
35	24	M	50	85	0	8
31	23.8	M	50	80	1	10
43	19.8	M	100	85	0	10
25	19.4	M	75	65	14	10
36	16	F	100	100	0	10
39	13.5	M		85	1	10

SF36 CH^a^, Change in Health

**Table 3:** Significant covariates of CFH_CSF _in normal subjects.

**Co-Variables with CRH_CSF_**	**rho**	**P**	**N**
Glucose @ 2 pm	0.987	0.0018	8
Sodium, mmol/L	-0.812	0.0079	9
Norepinephrine, baseline, pre-pain testing	0.790	0.035	7
Norepinephrine 15 min after bike exercise	0.918	0.0035	7
Heart rate, resting, pre-hand grip	0.796	0.018	8
Heart rate, hand grip, 1 min recovery	0.818	0.013	8
Heart rate, hand grip, 2 min recovery	0.747	0.033	8
Heart rate, hand grip, 3 min recovery	0.756	0.049	8
Heart rate, resting, pre-bike exercise	0.825	0.043	6
Heart rate, log, summed for the day	-0.810	0.015	8
Pain threshold for hyperthermia	-0.844	0.017	8
SF-36 Vitality	-0.838	0.0092	8
Self-Efficacy Scale – Manage Symptoms	-0.851	0.0073	8

**Table 4:** Significant covariates of CFH_CSF _and relevant statistics in males.

**Co-Variables with CRH_CSF_**	**Rho**	**P**	**N**
Respiratory Rate	0.953	0.0009	7
MIQ Vulnerability	-0.911	0.031	5
MFI Physical Functioning	-0.909	0.032	5
SF-36 Change in Health	-0.887	0.044	5
Self-efficacy – manage symptoms	-0.853	0.030	6

**Table 5:** Linear regression models of CRH_CSF_

**Components of 3 Linear Regression Models**	R^2 ^(adjusted R^2^) *	P **	DoF ***
CRH_CSF_, Glucose @ 2 pm, serum Sodium (mmol/L)	**0.979 **(0.958)	0.02	4
CRH_CSF_, Respiratory Rate, Resting Heart Rate	**0.963 **(0.939)	0.007	7
CRH_CSF_, SF-36 Vitality, MFI Physical Fatigue	**0.859 **(0.789)	0.02	6

* R^2 ^= expected variance; ** P = 2 tailed probability for the model; *** Degrees of freedom
